# Obstacle avoidance of physical, stereoscopic, and pictorial objects

**DOI:** 10.1007/s10055-025-01119-y

**Published:** 2025-03-01

**Authors:** Martin Giesel, Daniela Ruseva, Constanze Hesse

**Affiliations:** School of Psychology, University of Aberdeen, William Guild Building, Aberdeen, AB24 3FX UK

**Keywords:** Hand movements, Perception and action, Height perception, Distance perception, Binocular disparities, VR

## Abstract

Simulated environments, e.g., virtual or augmented reality environments, are becoming increasingly popular for the investigation and training of motor actions. Yet, so far it remains unclear if results of research and training in those environments transfer in the expected way to natural environments. Here, we investigated the types of visual cues that are required to ensure naturalistic hand movements in simulated environments. We compared obstacle avoidance of physical objects with obstacle avoidance of closely matched 2D and 3D images of the physical objects. Participants were asked to reach towards a target position without colliding with obstacles of varying height that were placed in the movement path. Using a pre-test post-test design, we tested obstacle avoidance for 2D and 3D images of obstacles both before and after exposure to the physical obstacles. Consistent with previous findings, we found that participants initially underestimated the magnitude differences between the obstacles, but after exposure to the physical obstacles avoidance performance for the 3D images became similar to performance for the physical obstacles. No such change was found for 2D images. Our findings highlight the importance of disparity cues for naturalistic motor actions in personal space. Furthermore, they suggest that the observed change in obstacle avoidance for 3D images resulted from a calibration of the disparity cues in the 3D images using an accurate estimate of the egocentric distance to the obstacles gained from the interaction with the physical obstacles.

## Introduction

We evolved in and adapted to a world with physical objects. We navigate around or towards them, avoid or intercept them, reach and grasp them, manipulate, modify, and use them, place them back or throw them away. To plan, program and guide these motor actions, our sensory systems (mostly visual, but also auditory, haptic and proprioceptive) gather information about the features (e.g., size) of these objects (intrinsic properties) and their location (extrinsic properties) relative to other objects (allocentric or exocentric distance) and to ourselves (egocentric distance). However, information about the objects in the world alone is not sufficient for the successful execution of motor actions. It also requires knowledge about how our body works (biomechanical factors), the required (biomechanical) effort in relation to the purpose of the movement, and associated risks/consequences of mistakes during the execution of actions (Hesse et al 2021a; Rosenbaum et al 2001; Schenk et al 2017; Utz et al 2015). In general, for everyday movements, this is mostly not a conscious process but integral part of the automatic programming and execution of movements. For more specialised and complicated movements, we might have to train to achieve this level of automatisation.

Extended reality environments (XR), e.g., virtual and augmented reality (VR/AR), have become increasingly popular for the investigation and training of human behaviour. One important reason for that is that they at least promise the possibility of full experimental control over environments that might, at some future point, approach the complexity of natural settings (Scarfe and Glennerster 2015, 2019; Snow and Culham 2021). However, so far it remains unclear if and how results of research and training of motor behaviour in XR environments transfer to natural environments (action fidelity). A reason for why they might not transfer in the expected way is that there are differences between XR and natural environments that might specifically affect the way motor behaviour is planned and executed (Giesel et al 2020; Brock et al 2023). Some of the differences are largely due to current technical limitations of XR devices. Current XR devices simulate environments that are perceptually less complex than their natural counterparts and potentially lack sources of veridical information that are typically available in natural environments for the planning and execution of actions. In that case, we must rely on the available, potentially inappropriately implemented, cues, for example to distance and size, resulting in increased visual uncertainty (Harris et al 2019).

One strategy to reduce this visual uncertainty and to achieve naturalistic behaviour in XR environments is trying to make these environments as complete as possible, i.e., to make all the sources of information present in natural environments available for the planning of actions in simulated environments (stimulus fidelity). However, while this might be desirable for some applications, such as entertainment, it has several drawbacks such as high computational demands.

Fortunately, there is evidence that action fidelity does not necessarily require stimulus fidelity (Stoffregen et al 2003). That is, the acquisition and transfer of motor skills can occur even when training environments lack realism. This is not necessarily surprising, as the human information system is capacity limited, meaning we rarely take advantage of the richness of all the visual information around us, but selectively process task-relevant visual cues (Schenk 2010). Furthermore, recent studies found that the accuracy of perception and actions in virtual environments improved after exposure to the corresponding natural environment (Hartle and Wilcox 2022; Interrante et al 2006), suggesting that it may be possible to pick-up task-relevant visual cues in the natural environment and use them to substitute missing cues in the simulated counterpart of that environment. However, it is, so far, unknown what kind of information is extracted from the natural environment, how it is applied to perception or actions in the simulated environment, and how similar those environments need to be for this to happen.

The aim of this work is to determine the minimal set of visual cues required to elicit naturalistic actions in XR environments, with the goal of informing the development of effective simulated training environments, and advancing our theoretical and practical understanding of a core function of vision: the guidance of actions. Research in the field of perception and action has for long been interested in elucidating the mechanisms guiding motor actions like reaching and grasping (Jeannerod 1981, 1984; Milner and Goodale 1995, 2006; Smeets and Brenner 1999). An important part of this research is the identification of the type of sensory (mostly visual) information that is used for programming and executing actions.

An influential theory, the perception-action model (PAM) by Milner and Goodale (1995, 2006) hypothesises that the visual information required for perception (vision-for-perception) and the visual information required for action (vision-for-action) are processed in anatomically distinct areas of the human brain (i.e., the ventral and dorsal pathways). The visual information in the two pathways is assumed to have different properties adapted to the purpose for which it is used. For example, one important requirement for vision-for-action is access to metrically accurate information about the egocentric distance of objects (vision-for-action), i.e., the distance of objects from ourselves or more specifically from that part of our body that interacts with the objects (i.e., effector), e.g., the fingers in a grasping task (Goodale 2008). In contrast, purely perceptual tasks subserved by vision-for-perception, e.g., verbal or manual estimations of size, are performed based on allocentric representations.

However, many have argued that the assumption of different neural structures is not necessary to explain differences between perception and action tasks nor do perception and action tasks necessarily differ (Franz and Gegenfurtner 2008; Kopiske et al 2016; Schenk et al 2011; Schenk and McIntosh 2010). Specifically, it has been argued that differences between perception and action may be better explained by different task requirements resulting in the use of different types of cues (Hesse et al 2021a; Smeets and Brenner 1999; Schenk et al 2017). For example, while most action tasks and some perceptual tasks require access to metrically accurate egocentric distance information, for most perceptual tasks allocentric distance information might be sufficient (Schenk 2006).

The perception of egocentric distances in XR has been investigated extensively using either verbal distance estimates, perceptual matching, or visually directed actions such as different variants of the blind walking task for distance perception in action space (for a review, see Renner et al 2013) and reaching or distance perception in the personal space (Bingham et al 2001; Gerig et al 2018). It frequently has been found that distance perception in XR is compressed, i.e., egocentric distances are underestimated compared to distances in natural environments (for a review, see Renner et al 2013). This might be the result of missing and/or conflicting information about egocentric distances. In natural settings, vergence (specifically horizontal vergence which is a disjunctive movement of the eyes, i.e., in- or outward rotation, that occurs when fixation changes between objects in different depth planes (Howard and Rogers 2008)) has been shown to provide accurate information about absolute egocentric distances at least in the personal space (Cutting and Vishton 1995; Mon-Williams and Tresilian 1999; Tresilian et al 1999). The findings by Linton (2020), however, question the usefulness of vergence as a cue to absolute distance. Due to the vergence-accommodation conflict this potential source of absolute distance information might be less reliable in XR (Harris et al 2019). Absolute distance information could also be derived from vertical disparities (Rogers and Bradshaw 1995) and motion parallax. However, there is so far little evidence that motion parallax is actually used to determine absolute distances (Creem-Regehr et al 2015; Hartle and Wilcox 2021).

Testing the perception of egocentric distances in virtual environments using a blind walking task, Interrante et al (2006) found that if the virtual environment was an exact replica of a physical environment, with which participants were already familiar, participants’ distance estimates were not compressed but similar to distance estimates in the physical environment. These findings were replicated several times (Interrante et al 2008; Phillips et al 2012; Steinicke et al 2010). A similar improvement of egocentric distance perception in virtual environments after prior exposure to the corresponding physical environment has been observed by Hartle and Wilcox (2022) using a manual depth estimation task. Interrante et al (2008) speculated that one possible explanation might be that in the physical environment participants have access to metrically accurate cues to egocentric distances which could then be used to calibrate the (incomplete/insufficient) cues to distance available in the corresponding (visually identical) virtual environment ("visual calibration hypothesis"). Identifying the perceptual and/or cognitive mechanisms underlying the observed improvement in distance perception could have important practical implications for the usage of XR environments in research and training.

Here, we aimed to systematically investigate the visual cues that are required for naturalistic behaviour in simulated environments and the effects of prior exposure to natural environments (containing all visual cues) on subsequent behaviour in simulated environments (lacking certain visual cues). Since we are mainly interested in actions in the personal space (i.e., reaching and grasping), we used obstacle avoidance tasks in which participants moved their hand over physical obstacles that varied in height or closely matched 2D or 3D images of these obstacles. We chose obstacle avoidance as a task because the absence of haptic feedback is not as relevant in obstacle avoidance as, for example, in grasping (e.g., Bingham et al 2007; Goodale et al 1994; Schenk 2012; Whitwell et al 2020), since the goal in obstacle avoidance is to avoid physical contact with the objects. In addition to obstacle avoidance, we also assessed participants’ perception of the height of the obstacles by using a manual height estimation task. Manual estimation tasks are frequently used in the perception-action literature (Hesse et al 2021b; Kopiske et al 2016; Westwood et al 2002) as the perceptual complement to action tasks (e.g., grasping). In these tasks, the participants do not directly interact with an object but, for example, adjust the distance between their thumb and index finger to indicate the size of objects that are grasped in the action task.

The rationale for our choice of tasks (obstacle avoidance and manual height estimation) and obstacles (physical, 3D and 2D images) was that they systematically differ in the number and type of informative cues to height and distance that they provide. For the physical objects, all cues to distance and size, binocular cues (binocular disparity), oculomotor cues (vergence and accommodation), motion-based cues (motion-parallax), and pictorial cues (e.g., occlusion, relative size, height in visual field, texture gradient) (e.g., Brenner and Smeets 2018) are available and might provide accurate information about the obstacles’ height and distance. For 3D images, binocular (horizontal) disparity and pictorial cues, and for 2D images only pictorial cues can provide reliable information about size and distance. Performances for the different types of obstacles will inform us about the relative importance of specific cues for the two different tasks.

We chose LEGO objects to build the physical obstacles because familiar size has been shown to influence distance perception and the programming of actions (McIntosh and Lashley 2008; Smeets et al 2022), and since LEGO is very familiar, most people should have an idea of the approximate size of LEGO blocks. We analysed how prior interactions with the physical obstacles influence subsequent obstacle avoidance and manual estimation with the 2D and 3D images of the obstacles to test effects of prior exposure to the physical obstacles on subsequent performance with virtual obstacles.

## Methods

We conducted four experiments. Table 1 provides an overview of the experiments. Experiments A–C consisted of three conditions. Experiment D had only one condition (see Section 3.4 for an explanation). The order of these conditions was always the same (pre-test post-test design). In the first condition, obstacles were presented as images (3D images in Experiments A and C and 2D images in Experiments B and D). In the second condition, the obstacles were physical (real) objects. In the third condition, the same images as in the first condition were presented, again. Participants performed the same task in all conditions of an experiment. In Experiments A and B, participants performed an obstacle avoidance task. In Experiments C and D, they performed a manual estimation task. In the following, we will refer to the first condition as the PRE condition, to the second condition as the REAL condition, and to the third condition as the POST condition.

**Table 1:** Obstacles and tasks used in the four experiments

Experiment		Obstacle		Task
	Pre	Real	Post	
A	3D image	Physical object	3D image	Obstacle avoidance
B	2D image	Physical object	2D image	Obstacle avoidance
C	3D image	Physical object	3D image	Manual estimation
D	2D image	-	-	Manual estimation

### Setup

**Fig. 1:**
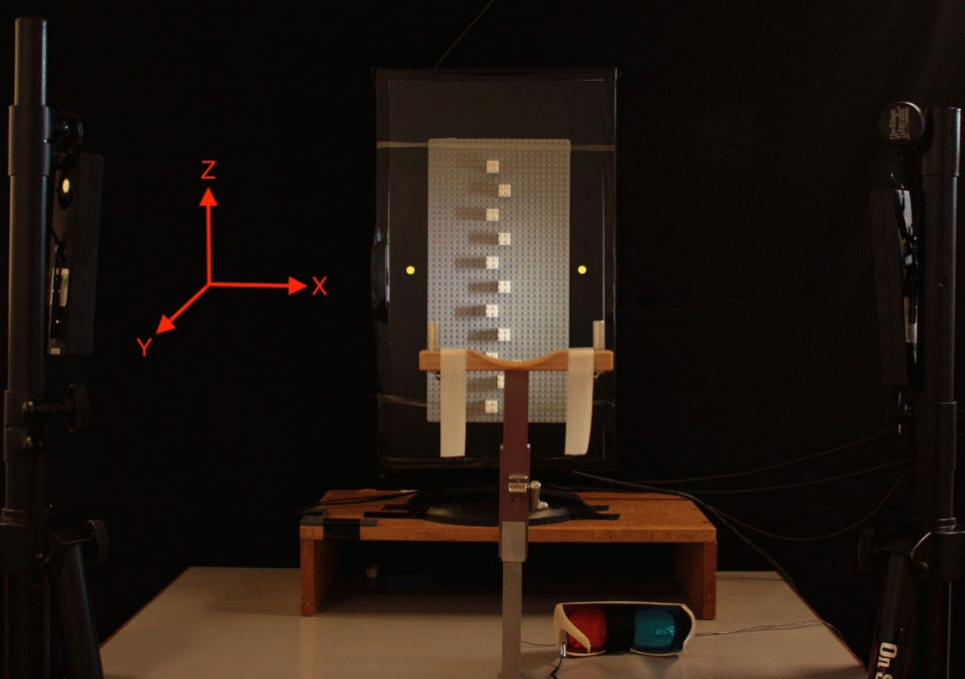
Setup with example physical obstacle (three blocks high) attached with transparent rubber bands to the surface of a monitor and anaglyph shutter glasses (lower right). The yellow dots to the left (start position) and right (target position) of the obstacle were only presented in the obstacle avoidance experiments (Experiments A and B). The rubber bands at the top and bottom remained in place even when the obstacles were presented as images on the monitor

Figure 1 shows the setup that was used for all experiments. A monitor (51.4 $\times$ 29 cm, 1920 $\times$ 1080 pixels) was placed on a table (61 $\times$ 91 $\times$ 74 cm). Obstacles were presented centred on the monitor either as images (PRE and POST conditions) or as physical objects (REAL condition). The physical objects were attached to the screen using transparent rubber bands (0.7 cm width) that were wrapped around the monitor at a distance of ±17 cm from the centre of the monitor. The rubber bands remained in place even when the stimuli were presented as images on the monitor. Participants sat on a height-adjustable chair in front of the monitor resting their head on a chin-rest in such a way that the centre of the monitor was at eye-level. The viewing distance to the monitor surface was 43 cm. The background of the monitor was always set to black. In all conditions of the obstacle avoidance experiments (Experiments A and B), there were two yellow dots (1 cm diameter) presented 2 cm to the left and right of the LEGO board. The left dot was the start position and the right dot the target position for the movement. The distance between start and target was 23.5 cm. Tripods with battery-operated LED lamps were placed on the left and right side of the table illuminating the obstacles in all conditions.

Participants’ hand movements were measured using an infra-red based Optotrak 3020 system (Northern Digital Incorporation, Waterloo, Ontario, Canada) with a sampling rate of 200 Hz tracking infra-red light emitting diodes attached to the participants’ hands. In Experiments A and B, one diode was attached to the nail of the right index finger. In Experiments C and D, one diode was attached to the nail of the right index finger and another to the nail of the right thumb.

Participants’ vision was occluded between trials and after movement onset using liquid crystal shutter goggles (PLATO Translucent Technologies, Toronto, Ontario (Milgram 1987)). Red (left eye) and cyan (right eye) filters were fitted into the shutter glasses (see Fig. 1). Note, participants wore the anaglyph shutter glasses in all conditions of all experiments independent of the obstacle type. The experiment was programmed in MATLAB (Mathworks, Natick, MA, USA) using the Optotrak Toolbox (Franz 2004) and the Psychtoolbox (Kleiner et al 2007; Pelli 1997; Brainard 1997).

### Obstacles

**Fig. 2:**
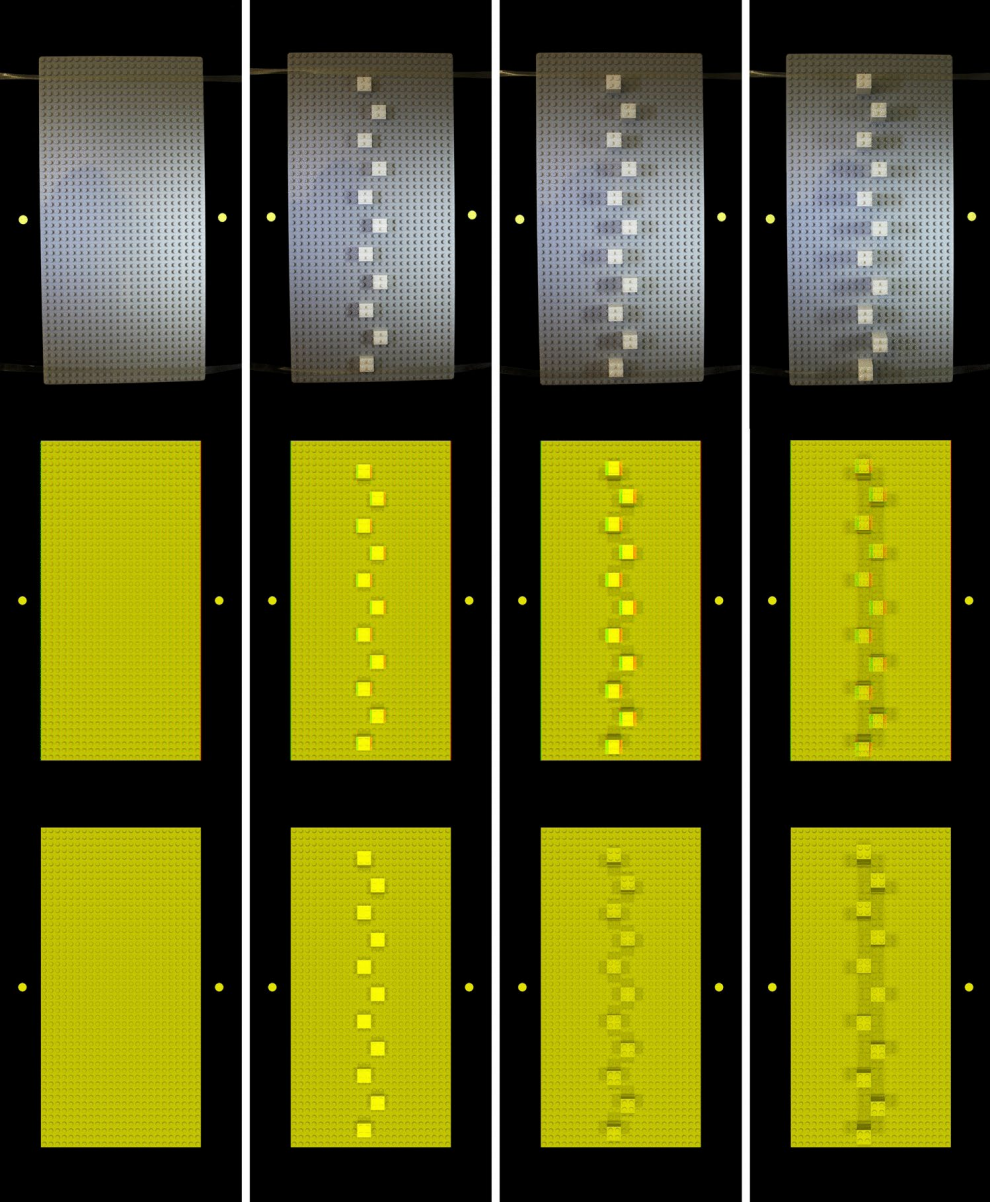
Obstacles used in the four experiments. Physical objects (first row), 3D images (second row), and 2D images (third row). The different heights of the obstacles are shown in columns (left to right: base board, 1, 2, 3 blocks high towers). The yellow dots indicate the start (left) and target positions (right) for the obstacle avoidance movement. They were only present in the obstacle avoidance experiments (Experiments A and B) not in the manual estimation experiments (Experiments C and D). The rubber-bands at the top and bottom of the monitor were also in place when the obstacles were images

Figure 2 shows the obstacles used in the four experiments.

#### Physical obstacles

The physical obstacles were constructed from LEGO material. The obstacles had four different heights. The lowest obstacle consisted of an empty grey LEGO base board (383 mm wide $\times$ 190 mm deep $\times$ 3.4 mm high). The other obstacles were created by adding 12 LEGO towers consisting of one, two, or three white square LEGO bricks (each brick had a width of 16 mm, height of 16 mm, and depth of 11.4 mm), respectively, on top of the base board. The blocks were placed alternately to the left or right of the vertical midline (to ensure better visibility of the full height of each obstacle) covering the entire length of the board. The vertical distance between towers was 16 mm. Table 2 shows the heights of the different physical obstacles.

Note, that because the LEGO boards were presented vertically in front of the monitor, the obstacles were pointing towards the participants. Consequently, obstacle height and distance varied along the same dimension (y-axis). In this case, obstacle height could be determined as the difference between the distances of the monitor surface and the top of the obstacles (allocentric distance). When in the following, we talk about obstacle height, we refer to this allocentric distance.

**Table 2:** Heights of the four physical obstacles. The studs on the bricks and the base board have a height of 1.8 mm, the plane part of the base board has a height of 1.6 mm. The bricks without the studs have a height of 9.6 mm

Physical obstacle	Height (mm)
Base board	3.4
1 brick	13.0
2 bricks	22.6
3 bricks	32.2

#### 3D images

The 3D images of the obstacles were presented as anaglyph stereo-images using the Psychtoolbox (Kleiner et al 2007; Pelli 1997; Brainard 1997). The stereo-images were rendered in Blender (Blender Online Community 2018). To create the images, 3D models of the four physical obstacles were build true to scale. The 3D models were illuminated in a way that created shadows that resembled the shadows of the physical obstacles when they were illuminated by the two lamps in the setup. Left eye and right eye images of the models were stereoscopically rendered using the "off-axis" method. The distance of the convergence plane was set to the viewing distance in the experiment (43 cm). Based on the average interocular distance, the inter-camera separation was set to 6.5 cm. This will result in the rendered binocular cues deviating from the correct binocular cues for each participant depending on the difference between each participant’s interocular distance and the average interocular distance. The sensor width of the cameras was set to 25 mm and the focal length to 25 mm. The resulting images had a size of 739 $\times$ 1660 pixels. We verified that the images of the obstacles covered the same area on the monitor as the physical obstacles when displayed on the monitor. The left and right eye images were displayed on the monitor as anaglyph (red-cyan) stereo images and viewed through anaglyph glasses.

#### 2D images

The 2D images were created in the same way as the 3D images, except that they were rendered from a centrally placed camera. The images were displayed on the monitor as if they had been anaglyph images.

### Procedure

At the beginning of each experimental session, participants were instructed about the task and provided written informed consent. The participants were asked whether they were familiar with LEGO, and whether they had played with LEGO. They did not see the physical LEGO obstacles before the second (REAL) condition of the experiments. Then the motion tracking marker(s) were placed on their finger(s), and they put on the shutter glasses. All movements were performed open-loop, i.e., there was no visual feedback once the obstacle avoidance movement or manual estimation had started. While in natural viewing conditions we rarely perform movements without access to visual feedback, the advantage of open-loop performance is that it gives us a clearer picture of how actions are planned based on the visual information available during the planning/preview stage without being modified by online corrections (i.e., corrections based on visual feedback during action execution). Especially, for simple ballistic movements and stable environments, as used in our obstacle avoidance experiments, the differences between open- and closed-loop conditions are small (e.g., Bruno et al 2016). Another reason to perform the obstacle avoidance task without visual feedback is to make the viewing conditions more similar to the manual estimation task. In the manual estimation task, participants are supposed to make estimates based on their height perception at a different spatial location. Thus, if both tasks were performed closed-loop, the direct comparison between finger and obstacle positions available in the obstacle avoidance task would not be possible in the manual estimation task. Lastly, the open-loop condition makes our experiments more similar to the majority of experiments investigating distance/depth perception in VR that used variants of the blind walking task. After the experiments, participants were asked to give a numerical estimate of the height of a single LEGO block. They were also informally questioned about the nature of the obstacles, and if and how often they had collided with the physical obstacles.

#### Obstacle avoidance (Experiments A and B)

The obstacle avoidance experiments consisted of three conditions. In the first condition (PRE), the obstacles were images of LEGO objects of different heights (3D images in Experiment A and 2D images in Experiment B). In the second condition (REAL), the obstacles were physical objects build from LEGO. In the third condition (POST), the same obstacles as in the first condition were presented, again. In each condition, there were obstacles of four different heights. The different obstacle heights were presented in pseudo-randomised order with 10 repetitions for each height. Each condition started with four practise trials.

At the beginning of a trial, participants’ right hand rested on the table, and the shutter glasses were closed. When the glasses opened, an auditory signal indicated to participants to lift the hand from the table and place their right index finger on the left yellow dot on the monitor (Fig. 1). The obstacles were also visible at this time. After correct placement of the index finger, there was a preview period of one second. Then, an auditory signal indicated that the participant should start to move to the yellow dot on the right side of the monitor without colliding with the obstacles. Specifically, the instructions were: "Please, lift your finger from the monitor, reach towards the target position without hitting the LEGO blocks, and briefly touch the right dot". As soon as their hand started to move (i.e., the 3D Euclidean distance between the index finger marker and the start position exceeded 25 mm), the shutter glasses closed. An auditory signal indicated if the target had been reached. Then participants placed the hand back on the resting position on the table. While participants were instructed to touch the target position, the actual criterion for a successful movement was that they had touched the monitor somewhere to the right of the obstacle board.

Participants could take a break between conditions. The procedure was the same for all conditions. In the REAL condition, the experimenter had to approach the monitor to exchange the obstacles in each trial. Participants were informed about the experimenter approaching them at a certain point during the experiment, but they were not told about the different types of obstacles (physical objects or images).

#### Manual estimation (Experiments C and D)

Analogous to the obstacle avoidance experiments, manual estimation in Experiment C consisted of three conditions (i.e., PRE, REAL, and POST). The obstacles in Experiment C were the same as those used in Experiment A (i.e., real obstacles and 3D images of the obstacles). Experiment D had only one condition. The obstacles in Experiment D were the same as those used in the first condition of Experiment B (i.e., 2D images of the obstacles). In the first condition (PRE), the obstacles were images of LEGO objects of different heights (3D images in Experiment C and 2D images in Experiment D). In the second condition (REAL), the obstacles were physical objects build from LEGO. In the third condition (POST), the same obstacles as in the first condition were presented, again. In each condition, there were obstacles of four different heights. The different obstacle heights were presented in pseudo-randomised order with 10 repetitions for each height.

At the beginning of a trial, participants’ right hand rested on the table, and the shutter glasses were closed. Then, the glasses opened for a one second preview period. An auditory signal indicated to the participants to lift their hand from the table and to adjust the distance between their right index finger and thumb so that it reflected the perceived height of the obstacles. As soon as the hand started to move (i.e., the 3D Euclidean distance between the index finger marker and the start position exceeded 25 mm), the shutter glasses closed. The participants verbally indicated to the experimenter when they were satisfied with their estimation adjustment, and the experimenter recorded the finger position via button press.

Participants could take a break between conditions. The procedure was the same for all conditions. In the REAL condition, the experimenter had to approach the monitor to exchange the obstacles in each trial. Participants were informed about this in advance, but they were not told about the different types of obstacles (physical objects or images).

### Participants

The criterions for the selection of participants for all experiments was that they had not participated in any of the other experiments (Experiments A, B, C, or D) or any of our previous experiments using similar obstacles and setup, had self-reported normal or correct-to-normal vision, normal colour vision and stereo vision. None of the participants reported visual discomfort or diplopia with the 3D images. All participants were naive as to the purpose of the experiment.

Experiment A: We collected data from 27 right-handed participants (15 female, age range = 18–39 years, mean (SD) age = 24.2 (±4.39) years). The experimental procedures were approved by the School of Psychology Ethics Committee (Ethics code: PEC/4841/2021/11). All observers gave written informed consent. They were compensated with £10 for their time.

Experiment B: We collected data from 18 right-handed participants (9 female, age range = 19–48 years, mean (SD) age = 27.9 (±8.34) years). The experimental procedures were approved by the School of Psychology Ethics Committee (Ethics code: PEC/4841/2021/11). All observers gave written informed consent. They were compensated with £10 for their time.

Experiment C: We collected data from 21 right-handed participants (15 female, age range = 19–39 years, mean (SD) age = 24.9 (±4.71) years). The experimental procedures were approved by the School of Psychology Ethics Committee (Ethics code: PEC/5039/2022/6). All observers gave written informed consent. They were compensated £10 for their time.

Experiment D: We collected data from 21 right-handed participants (15 female, age range = 18–42 years, mean (SD) age = 24.2 (±5.43) years). The experimental procedures were approved by the School of Psychology Ethics Committee (Ethics code: PEC/5127/2022/10). All observers gave written informed consent. They were compensated £4 for their time.

### Data analysis

In all experiments, the 3D trajectories for the finger(s) were filtered with a second-order Butterworth-filter with a low-pass cut-off frequency of 15 Hz. In the obstacle avoidance experiments, we rejected trials when the hand movement had started before the auditory start signal or the reaction time was shorter than 100 ms, the target criterion was not met (i.e., not touching the monitor surface to the right of the obstacle), or the marker was not visible to the motion tracker for longer durations during the movement resulting in missing data. Trials that during the experiments met these criteria were classified as an error and repeated later in the experiment at a random position. Additionally, 1 out of 3240 trials in Experiment A was excluded during data analysis. In Experiment A, eight out of 27 participants collided once with a physical obstacle, and three out of 18 participants collided once with a physical obstacle in Experiment B.

In the manual estimation experiments, we rejected trials when the hand movement started before the auditory start signal or the reaction time was shorter than 100 ms, no estimate was provide within 10 s, the estimation aperture changed before the aperture was recorded (i.e., aperture velocity larger than 0.03 m/s), or the markers were not visible at the time the aperture was recorded. Trials that during the experiments met these criteria were classified as an error and repeated later in the experiment at a random position. In Experiment C, one participant was excluded because they had misunderstood the instructions and provided estimates for the wrong dimension for some stimuli. Additionally, for the remaining participants, 22 out of 2400 trials were excluded during data analysis. In Experiment D, one participant was excluded because they did not follow the instructions. Additionally, 1 out of 800 trials was excluded during data analysis.

For the obstacle avoidance experiments, we analysed the trajectories in the direction perpendicular to the surface of the monitor (y-axis). The distance at which participants pass an obstacle can be thought of as representing an estimate of the perceived magnitude (height or distance) of the obstacles. Since it also incorporates an (usually constant) safety margin, it is not an accurate measure of the absolute magnitude of the obstacles but has been shown to accurately reflect magnitude differences between obstacles (e.g., Giesel et al 2020). For each trajectory, we determined the maximum y-value (peak movement distance from the monitor) reached between the start and end of the reaching trajectory. The start of the movement was determined by a distance criterion, i.e., the first time point where the distance between start position and index finger marker exceeded 25 mm. The end of the movement was determined as the first time point where the index finger had cleared the board in the horizontal direction (x-axis), i.e., the distance between the start position and the index finger marker was larger than 21 cm, and the y-distance between index finger and the monitor surface was smaller than 35 mm. For the manual estimation experiments, we analysed the estimation aperture, i.e., the 3D Euclidean distance between index finger and thumb markers at the time of the button press.

For all experiments and conditions, we computed linear regressions using a least-squares criterion for peak movement distance and height estimation data, respectively, separately for each participant. For each participant, a regression line was fitted through the 10 peak movement distance/height estimation data points for each obstacle height. For statistical analysis, peak movement distance and estimation data for Experiments A–C were analysed using 3 $\times$ 4 repeated-measures ANOVAs with the factors *presentation condition* (PRE vs REAL vs POST) and *obstacle height* (3.4, 13.0, 22.6, and 32.2 mm).

We expected that the sensitivity to magnitude differences between the obstacles differed between presentation conditions. This should result in a significant interaction between *presentation condition* and *obstacle height*. In this case, we evaluated the differences between presentation conditions by looking at the slopes and intercepts of the regression lines fitted to the data. The slopes and intercepts were analysed using one-way repeated-measures ANOVAs with the factor *presentation condition*. Manual height estimates in Experiment D were analysed using a one-way repeated-measures ANOVA with the factor *obstacle height*. If for the repeated-measures ANOVAs Mauchly’s Test of Sphericity indicated that the assumption of sphericity had been violated (p<.05), Greenhouse-Geisser corrected values were used (indicated by fractional degrees-of-freedom). If there was a significant main effect of the presentation conditions on slopes and intercepts in Experiments A–C, they were further analysed using two-sided paired-samples t-tests as well as their Bayesian equivalents. In Experiment D, one-sample t-tests were used to determine if the slopes were different from zero. For the Bayesian t-tests (JASP Team 2023; Morey and Rouder 2015; Rouder et al 2009), the (arbitrary) default Cauchy prior width of 0.707 was used. We report here only the Bayes factor. A more comprehensive analysis with plots of the prior and posterior distributions as well as Bayes factor robustness tests can be found on OSF. The robustness tests indicate that our findings are robust with respect to the choice of prior. Bayes factors measure the strength of evidence for one hypothesis relative to another hypothesis. For a two-sided Bayesian t-test, BF_10_ represents the Bayes factor supporting the alternative hypothesis compared to the null hypothesis, and BF_01_ is the Bayes factor supporting the null hypothesis compared to the alternative hypothesis. The relationship between them is: BF_10_=1/BF_01_. Bayes factors range continuously from 0 to $\infty$. A Bayes factor of 1 indicates that both hypotheses have equal support in the data. Following Jeffreys (1961), Bayes factors are often (arbitrarily) classified into three intervals. Bayes factors between 1 and 3 represent weak/anecdotal evidence, Bayes factors between 3 and 10 represent moderate evidence, and Bayes factors larger than 10 represent strong evidence. Statistical analysis was performed in Matlab and JASP (JASP Team 2023). JASP files for the data analysis performed here are available from the OSF (https://osf.io/6tf9r/).

## Results

**Fig. 3:**
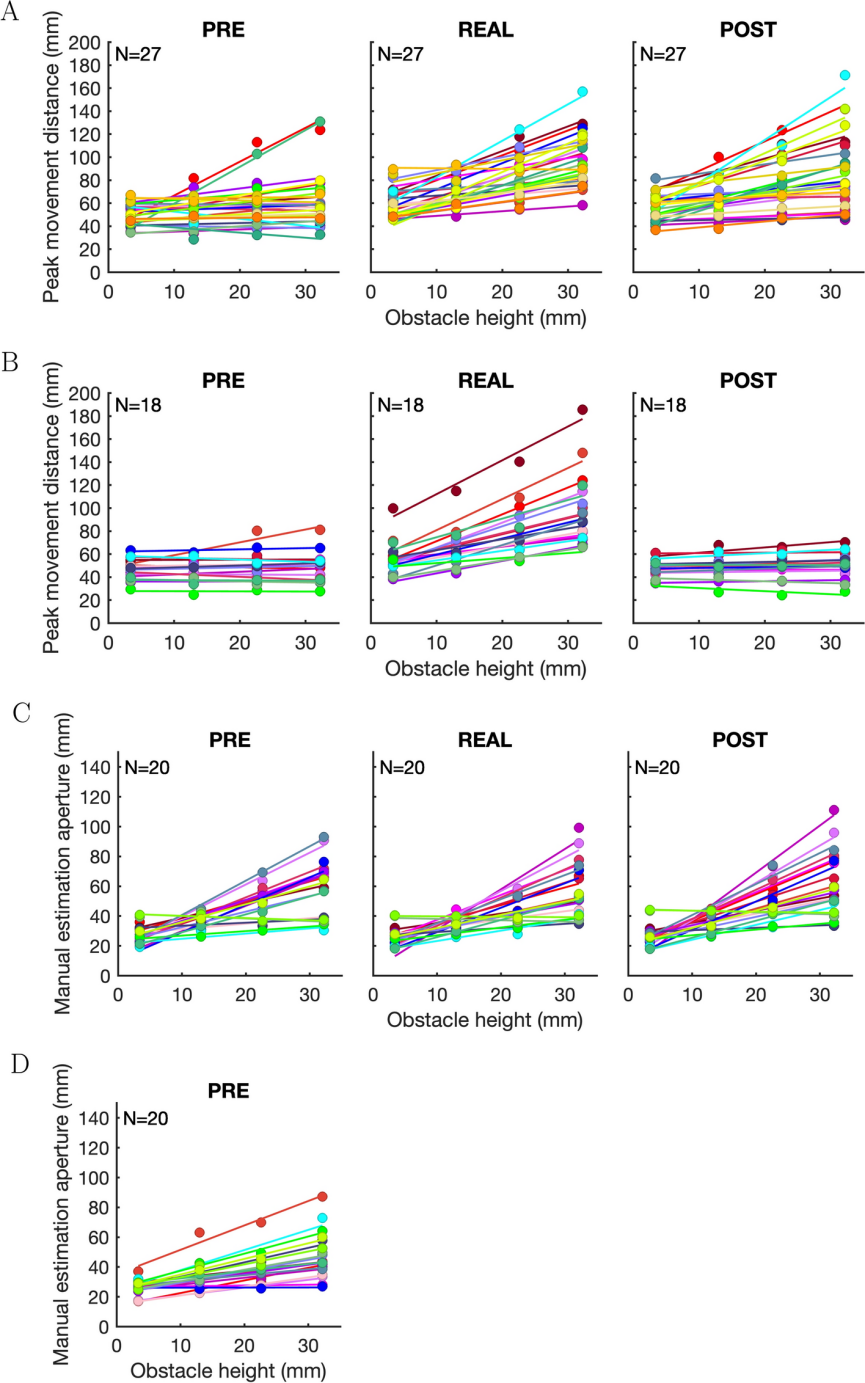
Mean peak movement distance data for the obstacle avoidance experiments **A** (real obstacles & 3D images) and **B** (real obstacles & 2D images), and mean height estimation aperture data for the manual estimation experiments **C** (real obstacles & 3D images) and **D** (2D images) for each participant with regression lines based on fits to the participants’ data. Each colour represents an individual participant. Note that the scaling of the y-axes differs between the obstacle avoidance (**A**, **B**) and the manual estimation (**C**, **D**) experiments

**Fig. 4:**
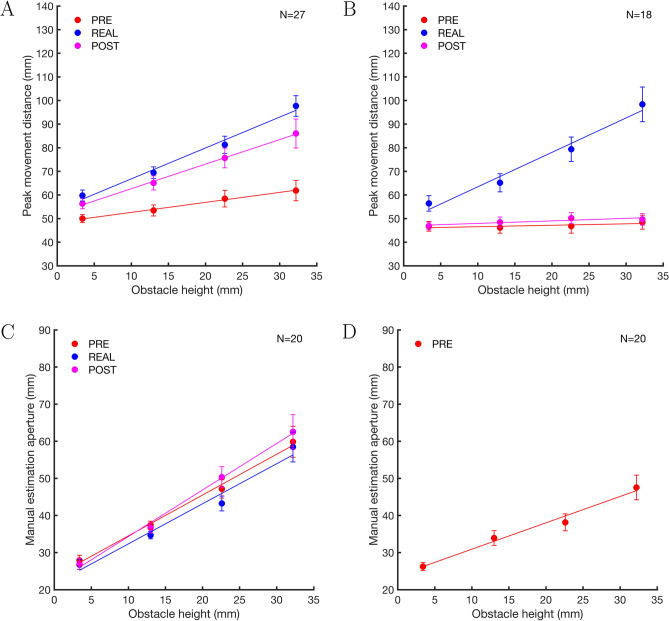
Peak movement distance data averaged over participants for the obstacle avoidance experiments **A** (real obstacles & 3D images) and **B** (real obstacles & 2D images), and height estimation aperture data averaged over participants for the manual estimation experiments **C** (real obstacles & 3D images) and **D** (2D images). Error bars show ± 1 SEM. Lines show regression lines based on the averaged regression parameters resulting from fits to individual participants’ data (see Fig. 3). PRE data are shown in red, REAL data in blue, and POST data in magenta. Note that the scaling of the y-axes differs between the obstacle avoidance (**A**, **B**) and the manual estimation (**C**, **D**) experiments

Figure 3 shows the mean peak movement distance and mean manual height estimates together with regression lines for each experiment, condition and participant. Figure 4 shows peak movement distance and manual height estimates for the different experiments and conditions averaged over participants, as well as regression lines based on slopes and intercepts averaged over participants. The results of the repeated-measures ANOVAs for peak movement distance and manual height estimation data are shown in Table 3.

**Table 3:** Results of the 3 $\times$ 4 repeated-measures ANOVAs with the factors *presentation condition* (PRE vs REAL vs POST) and *obstacle height* (3.4, 13.0, 22.6, and 32.2 mm) for Experiments A–C. Fractional degrees-of-freedom indicate Greenhouse-Geisser correction

Experiment	Cases	df	F	p	$\eta$_p_^2^
A	Presentation	2, 52	21.637	<.001	0.454
	Height	1.293, 33.629	45.810	<.001	0.638
	Presentation $\times$ Height	1.909, 49.644	10.599	<.001	0.290
B	Presentation	1.308, 22.231	41.170	<.001	0.708
	Height	1.534, 26.071	44.051	<.001	0.722
	Presentation $\times$ Height	2.154, 36.613	50.442	<.001	0.748
C	Presentation	1.396, 26.523	5.482	.018	0.224
	Height	1.104, 20.983	44.760	<.001	0.702
	Presentation $\times$ Height	2.026, 38.500	4.228	.021	0.182

### Experiment A

In Experiment A, we compared obstacle avoidance behaviour for 3D images of obstacles before and after obstacle avoidance for the corresponding physical obstacles. The results of Experiment A are shown in Figs. 3A and 4A. Note that for all obstacles the peak movement distance is considerably larger than the tallest obstacle. This additional distance is the safety margin that participants chose to safely pass the obstacles (in addition to the thickness of the index finger and the height of the marker sitting on top of the index finger). The peak movement distance data was analysed using a 3 $\times$ 4 repeated-measures ANOVA with the factors *presentation condition* (PRE vs REAL vs POST) and *obstacle height* (3.4, 13.0, 22.6, and 32.2 mm). Table 3 shows that there were significant main effects of *presentation condition* and *obstacle height*, and a significant interaction between *presentation condition* and *obstacle height*. The significant interaction between *presentation condition* and *obstacle height* might indicate that the sensitivity to magnitude differences between the obstacles differed between conditions. To analyse this in more detail, we looked at the slopes and intercepts of the regression lines fitted to each participant’s peak movement distance data (see Fig. 3A). Slopes and intercepts were analysed with one-way repeated-measures ANOVAs with the factor *presentation condition* (PRE vs REAL vs POST). In the following, average slopes and intercepts are given as M ± 1 SEM (between subjects). For slopes, there was a significant main effect of *presentation condition*, F(1.373,35.699)=12.308, p<.001, $\eta$_p_^2^=0.321. Pairwise comparisons showed a significant difference between the PRE (0.42 ± 0.16) and POST (1.04 ± 0.18) conditions, t(26)=–3.372, p_bonf_=.004, d=–0.717, and between the PRE and REAL (1.31 ± 0.16) conditions, t(26)=–4.838, p_bonf_<.001, d=–1.029. The difference between the REAL and POST conditions was not significant, t(26)=1.465, p_bonf_=.447, d=0.312. We further computed two-sided paired-samples Bayesian t-tests to compare the different presentation conditions with each other. The Bayes Factors showed that there was moderate support for the alternative hypothesis for PRE vs POST conditions, BF_10_=7.1, strong support for the alternative hypothesis for PRE vs REAL conditions, BF_10_=83, and only anecdotal support for the alternative hypothesis when comparing POST and REAL conditions, BF_10_=3.0.

The intercepts (PRE: 48.40 ± 1.89, REAL: 53.76 ± 2.71, POST: 52.27 ± 2.17) did not differ between presentation conditions, F(2,52)=2.737, p=.074, $\eta$_p_^2^=0.095. The slopes for the REAL and POST conditions were close to one and steeper than the slope for the PRE condition. A slope of one indicates that the peak movement distance accurately reflects the relative magnitude differences between the obstacles. After exposure to the physical obstacles, obstacle avoidance performance in the POST condition became more similar to performance in the REAL condition.

### Experiment B

In Experiment B, we compared obstacle avoidance behaviour for 2D images of obstacles before and after obstacle avoidance for the corresponding physical obstacles. The results of Experiment B are shown in Figs. 3B and 4B. The peak movement distance data was analysed using a 3 $\times$ 4 repeated-measures ANOVA with the factors *presentation condition* (PRE vs REAL vs POST) and *obstacle height* (3.4, 13.0, 22.6, and 32.2 mm).

Table 3 shows that there were significant main effects of *presentation condition*, and *obstacle height*, and a significant interaction between *presentation condition* and *obstacle height*. The significant interaction between *presentation condition* and *obstacle height* might indicate that sensitivity to magnitude differences between the obstacles differed between conditions. To analyse this in more detail, we looked at the slopes and intercepts of the regression lines fitted to each participant’s peak movement distance data (see Fig. 3B). Slopes and intercepts were analysed with one-way repeated-measures ANOVAs with the factor *presentation condition* (PRE vs REAL vs POST). For slopes, there was a significant main effect of *presentation condition*, F(1.387,23.574)=67.600, p<.001, $\eta$_p_^2^=0.799. Pairwise comparisons showed a significant difference between the PRE (0.06 ± 0.07) and REAL (1.46 ± 0.17) conditions, t(17)=–10.231, p_bonf_<.001, d=–3.061, and between the REAL and POST (0.11 ± 0.04) conditions, t(17)=9.900, p_bonf_<.001, d=2.962. The difference between the PRE and POST conditions was not significant, t(17)=–0.331, p_bonf_=1.0, d=–0.099. The two-sided paired-samples Bayesian t-tests showed that there was only anecdotal support for the alternative hypothesis for the comparison of the PRE and POST conditions, BF_10_=0.3, but strong support for the alternative hypothesis for both the comparison of the PRE and REAL conditions, BF_10_=123168, and the POST and REAL conditions, BF_10_=149419.

The intercepts (PRE: 45.95 ± 2.09, REAL: 48.89 ± 2.60, POST: 46.90 ± 1.64) did not differ between presentation conditions, F(1.300,22.106)=0.969, p=.359, $\eta$_p_^2^=0.054. The slopes for the PRE and POST conditions were both close to zero indicating that the peak movement distance was not affected by the different obstacle heights in the 2D images. There was no evidence for a change in performance after interaction with the physical obstacles.

### Experiment C

In Experiment C, we compared manual height estimation for 3D images of obstacles before and after height estimations for the corresponding physical obstacles. The results of Experiment C are shown in Figs. 3C and 4C (note that the manual height aperture data include the thickness of thumb and index finger as well as the height of the markers placed on those digits). The estimation grip aperture data was analysed using a 3 $\times$ 4 repeated-measures ANOVA with the factors *presentation condition* (PRE vs REAL vs POST) and *obstacle height* (3.4, 13.0, 22.6, and 32.2 mm).

Table 3 shows that there were significant main effects of *presentation condition* and *obstacle height*, and a significant interaction between *presentation condition* and *obstacle height*. The significant interaction might indicate that the sensitivity to magnitude differences between the obstacles differed between conditions. To analyse this in more detail, we looked at the slopes and intercepts of the regression lines fitted to each participants manual height estimation data (see Fig. 3C). Slopes and intercepts were analysed with one-way repeated-measures ANOVAs with the factor *presentation condition* (PRE vs REAL vs POST). The slopes for the PRE (1.10 ± 0.16), REAL (1.08 ± 0.16), and POST (1.25 ± 0.19) conditions were similar, and there was no significant main effect of *presentation condition*, F(1.409,26.770)=3.105, p$=$.077, $\eta$_p_^2^=0.140.

For intercepts, there was a significant main effect of *presentation condition*, F(2,38)=5.317, p$=$.009, $\eta$_p_^2^=0.219. Pairwise comparisons showed that the difference between the PRE (23.52 ± 1.79) and REAL (21.56 ± 1.91) conditions, t(19)=2.988, p_bonf_$=$.015, d=0.225, and between the PRE and POST (21.80 ± 2.13) conditions, t(19)=2.625, p_bonf_=.037, d=0.198, were significant. There was no significant difference between the REAL and POST conditions, t(19)=–0.363, p_bonf_=1.0, d=–0.027. The two-sided paired-samples Bayesian t-tests show moderate support for the alternative hypothesis for the comparison of the PRE and REAL conditions, BF_10_=7.9, and anecdotal support for the comparison of the PRE and POST conditions, BF_10_=2.0, and the POST and REAL conditions, BF_10_=0.3.

The analysis of the slopes shows that in all three conditions, participants were similar sensitive to the magnitude differences between the obstacles. There was no effect of the exposure to the physical obstacles on the sensitivity to magnitude differences. Intercepts were slightly higher in the PRE condition. In obstacle avoidance tasks, intercepts can be interpreted as safety margins (Giesel et al 2020), however, in manual estimation tasks it unclear what a change in intercepts signifies.

### Experiment D

In Experiment D, we measured manual height estimation for 2D images of obstacles. We only tested the PRE condition because we did not expect to find a change in height estimation for 2D images after exposure to the physical obstacles since we found no such effect for the 3D images in Experiment C. However, we wanted to find out if participants were able to perceive height difference in the 2D images. The results of Experiment D are shown in Figs. 3D and 4D. The estimation grip aperture data was analysed using a one-way repeated-measures ANOVA with the factor *obstacle height* (3.4, 13.0, 22.6, and 32.2 mm). There was a significant main effect of *obstacle height*, F(1.339,25.448)=54.778, p<.001, $\eta$_p_^2^=0.742. The mean intercept was 23.82 ± 1.07, and the mean slope was 0.71 ± 0.09. A one-sample t-test showed that the slopes were significantly different from zero, t(19)=8.038, p<.001, d=1.797. A one-sample Bayesian t-test shows strong support for the alternative hypothesis that the slope is different from zero, BF_10_=98521. Although, the slope was lower than one, even with only pictorial cues to obstacle height, participants were still surprisingly good at estimating the different obstacle heights.

## Discussion

The aim of this series of experiments was twofold: Firstly, we aimed to identify the visual cues required to perform accurate actions in simulated environments and determine if the cues required vary depending on the type of task performed (i.e., action vs. perception tasks). Secondly, we wanted to test if and how prior exposure to natural environments affects actions performed in simulated environments (i.e., "visual calibration hypothesis").

We found that performance in action (i.e., obstacle avoidance) and perception (i.e., manual height estimation) tasks differed in the presence of virtual obstacles (i.e., 3D and 2D images) but was comparable for physical obstacles. Specifically, participants (initially) underestimated the magnitude differences between obstacles in the action task when the obstacles were 3D images but perceived them accurately in the corresponding perceptual task. Interestingly, 2D images of the obstacles did not trigger a height dependent obstacle avoidance movement despite the perceptual task indicating that participants were able to perceive some height differences between obstacles in those images. With regard to the effects of prior exposure, we found that after interacting with the physical obstacles, actions with the 3D images resembled those with the physical obstacles.

Regarding the effects of prior exposure, our main finding in Experiment A was that in an obstacle avoidance task we observed that interactions with the physical obstacles changed avoidance behaviour in a subsequent obstacle avoidance task with 3D images of the same obstacles compared to behaviour before exposure to the physical obstacles. Before exposure to the physical obstacles, we found that the magnitude differences between the obstacles were underestimated resulting in slopes of the regression lines fitted to the peak movement distance data lower than one. This underestimation is consistent with the majority of findings for blind walking in virtual environments (Renner et al 2013). After exposure to the physical obstacles, obstacle avoidance behaviour for the 3D images of the obstacles became similar to obstacle avoidance behaviour for the physical obstacles. The observed change after exposure to the physical obstacles was an increase in the steepness of the regression line fitted to the data, indicating that obstacle avoidance behaviour more accurately reflected the magnitude differences between the obstacles. It seems unlikely that the change in performance between PRE and POST conditions was simply due to a practise effect because there was never any feedback regarding participants’ performance, and since, obviously, images of obstacles provide no haptic feedback, moving the hand at any distance away from the monitor would have been a safe distance. These findings align with the findings by Interrante et al (2006) and others for blind walking in virtual environments after exposure to the physical environment.

Experiments B-D help us to better understand what might have caused the change in obstacle avoidance behaviour after exposure to the physical obstacles in Experiment A. In Experiment B, we found no difference in obstacle avoidance behaviour for the 2D images before and after exposure to the physical objects. The flat regression lines in the PRE and POST conditions indicate that the pictorial cues to height and distance available in the 2D images did not affect the peak distance of the avoidance movement at all. This is consistent with previous findings about the role of pictorial cues in reaching and grasping (e.g., Ozana et al 2018). Moreover, it indicates that the change between PRE and POST conditions in Experiment A is likely not the result of participants in some way pantomiming their movements, i.e., relying on a stored perceptual representation of previously seen objects (Milner and Goodale 1995, 2006), for the physical obstacles in the POST condition. If the change in behaviour was entirely a cognitive effect based on making the connection between the two environments, then there is no reason why it should not also have had an effect on behaviour with 2D images. The behaviour for the 2D images is of course entirely sensible. Participants move their fingers at a constant distance away from the monitor surface to reach the target because there was no variation in height or distance between the 2D obstacles. However, the same also applies to the 3D images in Experiment A. Yet, for the 3D images the distances of the depicted obstacles affected the avoidance movements already before exposure to the physical obstacles. The informal questioning of the participants after the experiment showed that they were aware that the 3D and 2D images were in fact images. Together, Experiment A and B point towards the importance of the presence of binocular disparity cues for the accurate execution of actions (Bradshaw et al 2004).

In Experiments C and D, we asked participants to estimate the height of the obstacles depicted in the 3D and 2D images before and after exposure to the physical obstacles. Participants very accurately estimated the height differences for the physical objects and the 3D images (Experiment C) already in the PRE condition (slope of the regression line close to one). There was no further change in the POST condition. The findings of Experiment C confirm that the 3D images contained the cues necessary for accurate height estimates and that they were used for manual height estimates. This also suggests that the change in the obstacle avoidance behaviour in Experiment A is likely not the result of an increased sensitivity to height differences. Height estimates for the 2D images of the obstacles (Experiment D) reflected height differences between them less accurately than for the 3D images but the slopes of the regression lines were different from zero indicating that the 2D images contained pictorial cues to obstacle height that influenced perceptual height estimates but not obstacle avoidance movements (see Experiment B). The findings of Experiments C and D support the idea that action tasks, like obstacle avoidance, and perceptual tasks, like manual height estimation, might either be processed differently (Milner and Goodale 1995, 2006) or rely on different cues (Hesse et al 2021a; Smeets and Brenner 1999; Schenk et al 2017). The notion that cue use differs between perception and action tasks is supported by findings of Kunz et al (2009) who observed that in contrast to blind walking, verbal distance estimates were affected by the quality of the rendering of the virtual environment. A high quality rendering of an environment might contain more and more effective pictorial cues to distance and size than a low quality rendering and this might improve performance in perceptual estimation tasks. For action tasks, however, the quality of pictorial cues might be largely irrelevant because they provide little information about absolute egocentric distances.

As mentioned above neither the 2D nor 3D images of obstacles represent actual obstacles for actions. Participants could have made similar movements in both conditions. Yet, we found clear differences in participants’ behaviour for the two types of pictorial obstacles. One could speculate that by exposing participants to the physical obstacles they may have acquired some kind of internal representation of these obstacles based on which they then perform avoidance movements in the POST condition. Yet, two findings speak against this: firstly, participants’ movements are already affected by obstacle height in the PRE condition for 3D pictorial obstacles (albeit to a lesser extent than in the REAL condition) and secondly, participants’ movements remain unaffected by obstacle height for 2D pictorial obstacles even after exposure to the physical obstacles. As participants were exposed to the same physical obstacles in both the 3D and 2D obstacle avoidance experiments (Experiments A and B), it would be difficult to explain how these different behaviours could be caused by the same internal representation acquired in the REAL condition. Rather, our results suggest that obstacle avoidance behaviour is largely driven by the available information and task requirements. While we derived our experiments, predictions, and interpretations from the perception-action framework, we would like to acknowledge that they are also consistent with the ecological approach to perception and action (Gibson 1979). Within this approach the improvement in obstacle avoidance performance for 3D pictorial obstacles after exposure to the physical obstacles could be explained as perceptual learning based on differentiation whereby participants get better at identifying and discriminating relevant information in the sensory input (Gibson 2000; Segundo-Ortin and Raja 2024). Note, however, that the performance differences between 2D and 3D pictorial obstacles still indicate that there is specific relevant information for obstacle avoidance behaviour that is available in the 3D condition but not in the 2D condition (see below). The evaluation of the two different theoretical approaches to perception (i.e. “indirect” and “direct” theories of perception) is a complex, and in many aspects philosophical, issue that goes beyond the scope of this paper.

Many evaluations of the quality of XR focus on the experience of presence, or specifically for VR environments, the "place illusion" which refers to a "... strong illusion of being in a place in spite of the sure knowledge that you are not there" (Slater 2009, p. 3551). Investigations of the strength of presence in a given XR environment have mostly relied on questionnaires (e.g., Slater et al 1995; Usoh et al 1999), and measures of physiological activation, e.g., heart rate or skin conductance (e.g., Meehan et al 2002). However, if we want to use XR to investigate or train behaviour in natural environments these measurements might be too broad and unspecific. We actually have to directly compare movements between closely matched natural and XR environments (e.g., Brock et al 2023; Giesel et al 2020). Our findings might indicate that, as suggested by Slater (2009), different definitions of presence might be required depending on the task.

Our results have some similarity with those of Hartle and Wilcox (2022) who set out to compare depth estimates for physical objects, and closely matched pictorial objects presented in a stereoscope and in a head-mounted VR display. Their results showed that participants who had completed the distance estimation task first with the physical objects, provided more accurate distance estimates for the subsequently seen virtual objects than those who had started with the pictorial objects. In the framework of perception and action research, their task is a perceptual task (manual estimation). That they found an improvement effect for manual distance estimation after exposure to the physical objects, which we did not observe for manual height estimation, suggests that the differentiation between perception and action might not be the relevant criterion here. Rather, it might depend on whether a task requires absolute egocentric distances or not (see also Schenk 2006). For example, accurate height estimates for the 3D obstacles in Experiment C do not necessarily require knowledge of the absolute egocentric distance of the (top of the) obstacles from the participant but only the allocentric distance between the top of the obstacles and the baseplate. Relative distance information can be derived from disparity cues and, especially for objects placed in the personal space, disparity information is quite accurate for size estimates (Richards 2009; Volcic et al 2013).

Our findings indicate that binocular disparities are a necessary but not sufficient cue for the accurate execution of actions. To achieve correspondence between natural actions and actions in XR environments, disparities have to be calibrated by cues that provide access to absolute distances as hypothesised by Interrante et al (2008) ("visual calibration hypothesis"). In our experiments, participants might have picked up these cues when interacting with the physical objects. The exact mechanism of how this calibration of disparities is achieved remains currently unclear.

One could argue that the restricted viewing conditions in our experiments may not adequately represent natural viewing conditions where participants are free to move. Self-generated motion has been shown to support precise and accurate perception of egocentric distances (e.g., Mantel et al 2015). However, what speaks against a crucial role of self-generated movement in simple worktop-based hand movement tasks, is the observation that even if head movements are completely unrestricted, participants do not tend to substantially move their head, i.e., observed head movements do usually not exceed 2$-$2.5 cm (Dijkerman et al 1999; Marotta et al 1995). Thus, it has been argued that when binocular and pictorial depth cues are available, motion parallax is not a major source of depth information for tasks like those used in our experiments. Furthermore, proprioceptive information, which has also been argued to be a source of metric distance information for action tasks (Rossetti et al 1995; Van Beers et al 1999), was the same in all our obstacle avoidance tasks. Independent of whether the objects were physical objects, 3D or 2D images, there always was a (physical) monitor present on which participants placed their finger. Changes in proprioceptive information can therefore not explain the differences we found in obstacle avoidance performance for the different types of stimuli. The monitor could also have provided a visual reference for the obstacle avoidance task, but this also seems not to have been used by participants.

Almost all of our participants reported being familiar with LEGO. In line with the findings by Interrante et al (2008), this indicates that at least for the performance of actions, familiar size is likely not a sufficient cue to determine egocentric distances. Given that our informal assessment of participants verbal estimates of the height of a single LEGO brick varied widely, it also seems unlikely that familiar size had a strong effect on manual height estimates. Another interesting question is how persistent this effect of exposure to the physical environment might be. An anecdotal observation suggests that the effect might be quite persistent, since we first observed it with a participant who had previously participated in a different experiment using the same type of LEGO objects.

Finally, our findings along with those by Hartle and Wilcox (2022) and Interrante et al (2006) have methodological implications. As systematically investigated by Keefe and Watt (2009), the order in which we test conditions that provide different types or amounts of information about task relevant features of the stimuli matters. Especially, if we follow the "tear-down approach" advocated by Snow and Culham (2021), we have to be aware that starting with the most complete stimulus might influence (improve) performance for subsequently viewed less complex stimuli.

## Conclusion

We investigated obstacle avoidance and manual height estimation for physical obstacles that varied in height and closely matched 2D and 3D images of these obstacles. We were interested in identifying the visual cues that are required for naturalistic behaviour in simulated environments and in finding out if and how prior exposure to the physical obstacles influenced performance for virtual obstacles. We found that after exposure to the physical obstacles, obstacle avoidance performance for 3D images of the obstacles became similar to obstacles avoidance for the physical obstacles. Avoidance behaviour for 2D images of the obstacles was not influenced by the height of the depicted obstacles. This did not change after exposure to the physical obstacles. Manual height estimates for the 3D images of the obstacles were already accurate before exposure to the physical obstacles. Our finding highlight the importance of the presence of binocular disparity cues for naturalistic motor actions. The increased accuracy after exposure to the physical obstacles supports the hypothesis that during interaction with the physical obstacles participants picked up information about accurate egocentric distances which might have been used to calibrate the disparity cues. Our findings further emphasise that different types of tasks might require different types of visual information about the properties of the environment and the objects in it (e.g., allocentric vs egocentric distance information). This should be considered when evaluating the suitability of XR environments for different types of tasks. Specifically, purely perceptual evaluations might not be sufficient to ensure action fidelity.

## Data Availability

All data and statistical analyses presented here are available from the OSF with this link: https://osf.io/6tf9r/.
